# Visual Perception and Pre-Attentive Attributes in Oncological Data Visualisation

**DOI:** 10.3390/bioengineering12070782

**Published:** 2025-07-18

**Authors:** Roberta Fusco, Vincenza Granata, Sergio Venanzio Setola, Davide Pupo, Teresa Petrosino, Ciro Paolo Lamanna, Mimma Castaldo, Maria Giovanna Riga, Michele A. Karaboue, Francesco Izzo, Antonella Petrillo

**Affiliations:** 1 Division of Radiology, Istituto Nazionale Tumori IRCCS Fondazione Pascale—IRCCS di Napoli, 80131 Naples, Italy; r.fusco@istitutotumori.na.it (R.F.); s.setola@istitutotumori.na.it (S.V.S.); davide.pupo@istitutotumori.na.it (D.P.); t.petrosino@istitutotumori.na.it (T.P.); cm.lamanna@istitutotumori.na.it (C.P.L.); a.petrillo@istitutotumori.na.it (A.P.); 2 Unit of “Progettazione e Manutenzione Edile ed impianti”, Istituto Nazionale Tumori IRCCS Fondazione Pascale—IRCCS di Napoli, 80131 Naples, Italy; m.castaldo@istitutotumori.na.it; 3 Department of Radiology, University of Padova, 35122 Padova, Italy; mariagiovanna.riga@gmail.com; 4 Department of Clinical and Experimental Medicine, University of Foggia, 71122 Foggia, Italy; michelekaraboue@gmail.com; 5 Division of Epatobiliary Surgical Oncology, Istituto Nazionale Tumori IRCCS Fondazione Pascale—IRCCS di Napoli, 80131 Naples, Italy; f.izzo@istitutotumori.na.it

**Keywords:** visual perception, pre-attentive attributes, data visualisation, oncology

## Abstract

In the era of precision medicine, effective data visualisation plays a pivotal role in supporting clinical decision-making by translating complex, multidimensional datasets into intuitive and actionable insights. This paper explores the foundational principles of visual perception, with a specific focus on pre-attentive attributes such as colour, shape, size, orientation, and spatial position, which are processed automatically by the human visual system. Drawing from cognitive psychology and perceptual science, we demonstrate how these attributes can enhance the clarity and usability of medical visualisations, reducing cognitive load and improving interpretive speed in high-stakes clinical environments. Through detailed case studies and visual examples, particularly within the field of oncology, we highlight best practices and common pitfalls in the design of dashboards, nomograms, and interactive platforms. We further examine the integration of advanced tools—such as genomic heatmaps and temporal timelines—into multidisciplinary workflows to support personalised care. Our findings underscore that visually intelligent design is not merely an aesthetic concern but a critical factor in clinical safety, efficiency, and communication, advocating for user-centred and evidence-based approaches in the development of health data interfaces.

## 1. Introduction

Visual perception is the cognitive process through which stimuli captured by the eyes are interpreted and transformed into meaningful information. In the context of data visualisation, a deep understanding of how visual perception operates is essential, as it enables the creation of graphics that effectively leverage both the strengths and limitations of the human visual system to communicate information in a clear and efficient manner. Empirical evidence suggests that well-designed visualisations can exploit the high sensory bandwidth of the visual modality to rapidly convey complex datasets. In other words, when perceptual principles are integrated into the design process, visualisations can significantly enhance both the accuracy and the amount of information that users are able to process intuitively [1]. Design choices that align with natural visual mechanisms facilitate the immediate recognition of patterns, trends, and outliers, minimising cognitive effort and maximising interpretative speed.

One critical element in this process is iconic memory, a form of ultra-short-term visual storage that persists for a few hundred milliseconds. Iconic memory enables parallel processing of the visual field, allowing individuals to detect salient visual features without requiring focused attention. For example, a brightly coloured road sign appearing in the periphery of the visual field is immediately registered, even without conscious effort. This pre-attentive scanning mechanism, shaped by evolutionary pressures (e.g., detecting predators or threats in the environment), remains active in modern cognitive function [2]. In the context of data visualisation, this means that certain elementary visual features—such as colour, shape, or spatial location—are processed automatically and can be used to direct attention to critical elements of a visual display before conscious analysis begins.

In clinical applications, these principles have direct implications. A well-structured chart or dashboard can convey essential patient status information or epidemiological trends almost at a glance, supporting timely and informed decision-making. The historical importance of visualisation in healthcare is well documented: in the 19th century, John Snow’s cholera maps enabled the identification of transmission sources, while Florence Nightingale’s statistical diagrams demonstrated the impact of hospital sanitation on mortality rates [2]. In contemporary practice, the proliferation of electronic health records (EHRs) has highlighted how poorly designed interfaces can increase the cognitive load on clinicians, leading to delayed interpretation and errors. Conversely, well-designed data displays, grounded in perceptual science, can reduce mental strain and improve accuracy in clinical judgement [3].

Thus, applying principles of visual perception and pre-attentive processing is not merely an aesthetic concern but a key element in the design of effective clinical dashboards, epidemiological charts, and health communication tools. Leveraging these mechanisms enhances usability, accelerates information uptake, and ultimately supports better clinical outcomes.

## 2. Pre-Attentive Attributes and Their Role in Visual Perception

In the domain of visual perception, pre-attentive attributes are defined as those visual features that are processed rapidly and automatically by the human visual system prior to the engagement of conscious attention. These properties are termed “pre-attentive” because their processing precedes the phase of focal, deliberate observation. A widely accepted heuristic is that any visual task completed in less than approximately 200 ms is considered pre-attentive [2]. This duration roughly corresponds to the minimum time required to initiate an eye movement. Consequently, if an observer detects an item in an image within that time frame, it implies that the detection occurred in a single glance, without the need for point-by-point inspection [2,3,4].

This capability is made possible by the parallel processing mechanisms of the low-level visual system, which simultaneously extract certain elementary features across the visual field [1]. These basic visual attributes include colour, shape, size, orientation, and spatial position. When a visual element in a graph uniquely possesses one of these attributes—i.e., it differs from surrounding elements (“distractors”)—it tends to stand out immediately, attracting the observer’s attention without effort.

This effect is further supported by the activity of iconic memory, a transient form of visual sensory memory that briefly retains a high-resolution “snapshot” of the visual scene. It allows salient patterns or anomalies to be detected almost instantaneously, without conscious deliberation.

Pre-attentive attributes can be grouped into well-documented categories, although slight variations exist in the literature. The most commonly recognised include the following:

- Colour (hue and intensity): Differences in colour, such as a red item among blue ones, represent one of the most effective pre-attentive signals. Variations in brightness or saturation can also elicit rapid detection [5].
- Shape: Distinctive shapes (e.g., a circle among squares) are easily recognisable without focused attention. Psychologists have also identified features such as closure (e.g., solid vs. hollow shapes) as pre-attentively processed [6].
- Size: Large discrepancies in size between objects, such as a significantly larger symbol, tend to attract immediate attention. Symbol size in charts is often interpreted at a pre-attentive level [1,2,3,4].
- Orientation: An item oriented differently (e.g., a tilted line amid vertical lines) is readily noticed. Simple orientations—vertical, horizontal, or diagonal—are typically processed early [1].
- Spatial position: The relative location of elements in space is a powerful perceptual cue. Isolated or misaligned items, or those deviating from an expected layout, are immediately perceived as distinct. Furthermore, position on a common scale (such as bar height in a histogram) is interpreted efficiently by the visual system.

Pre-attentive perception relies on rapid, automatic biological mechanisms that occur early in visual processing, before conscious attention or deliberate reasoning is activated. These processes occur in the retina and primary visual cortex (particularly areas V1 and V2), where specialised neurons respond selectively to basic visual features. For example, certain retinal cells and cortical pathways are tuned to detect chromatic contrast (colour), contours and angles (orientation), spatial extent (size), and the position of objects.

Evolution has favoured these perceptual channels because they allow rapid responses to potentially relevant stimuli, such as a sudden movement or a visually distinct anomaly in the environment. The most effective pre-attentive features are those that activate separate, dedicated neural pathways: colour stimulates cones and parvocellular flow; size activates spatial-scale neurons; shape is recognised through elementary geometric configurations; orientation is detected by orientation-selective cells; and the location is mapped onto the retinotopic organisation of the visual cortex. These attributes are perceptually relevant because each of them triggers the so-called “pop-out effect”: an object that differs from its surroundings along one of these dimensions is perceived immediately, even without focused attention. This is precisely why they are widely used in visualisation systems, including clinical dashboards and medical interfaces, to guide the user’s attention to the most critical data without overloading working memory.

Other attributes, such as texture, curvature, enclosure (e.g., an object outlined by a border), and motion (in dynamic visualisations), have also been shown to function pre-attentively in specific contexts [1]. However, in two-dimensional static graphics, the five aforementioned attributes, colour, shape, size, orientation, and position, remain the most widely utilised and perceptually salient.

The significance of pre-attentive attributes in graphical communication lies in their ability to convey information instantly and subconsciously. Since these attributes are processed in under half a second and without cognitive load, their strategic use can greatly enhance the user’s ability to scan and interpret complex visual displays. For instance, highlighting a data point with a contrasting hue can dramatically reduce the time needed to locate it within a crowded chart. As Healey and colleagues note, an effectively chosen pre-attentive attribute can guide the viewer’s attention directly to a visual “target”, bypassing the need for exhaustive search [7]. Moreover, because pre-attentive processing is largely automatic, it imposes minimal cognitive burden, preserving mental resources for the interpretation of more complex or abstract relationships within the data.

In summary, pre-attentive features are powerful instruments in the design of intuitive and efficient visualisations. They support the creation of visual hierarchies, enabling designers to indicate relative importance (e.g., through size or colour) and to structure the flow of visual attention throughout the display (Table 1). However, these attributes must be used with care: excessive or conflicting use of pre-attentive cues may lead to confusion or visual clutter, undermining the clarity of the message, as will be discussed in the following section.

**Figure 1:**
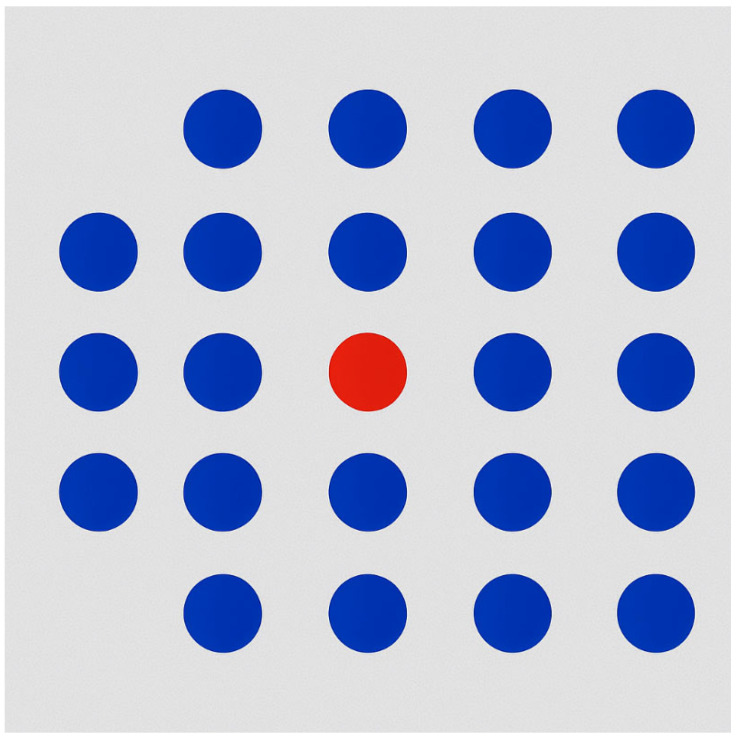
A set of shapes that are all the same (blue circles) contains an element with a different colour (red circle). The red circle is immediately noticed due to the pre-attentive attribute of colour.

As illustrated in Figure 1, a simple abstract example demonstrates the effectiveness of colour as a pre-attentive attribute. The figure displays a grid of uniformly coloured blue circles, within which a single red circle is placed. The red element is immediately perceived by the observer, without the need for a serial or conscious search through all the elements. This occurs because the difference in hue activates rapid visual discrimination mechanisms: the contrast between the red target and the blue distractors creates a salient visual signal, which is rapidly processed via iconic memory and the early visual cortex. Such perceptual saliency enables users to detect the target “at a glance”, typically within approximately 200 ms, which corresponds to the time window of pre-attentive visual processing, occurring before an eye movement or focused attention is engaged [5].

In the context of data visualisation, if the aim is to highlight a specific data point among many, using a distinctive colour is a robust and efficient method to direct attention. Similar perceptual effects can be achieved using other pre-attentive features [6]. For instance, a circle among squares stands out due to shape variation; a larger object amidst smaller ones is immediately noticeable due to size contrast.

The key principle is feature uniqueness: only the item of interest should possess the distinguishing attribute, while all others remain visually homogeneous. This asymmetry triggers a unique perceptual response, enabling rapid, low-effort detection.

**Figure 2:**
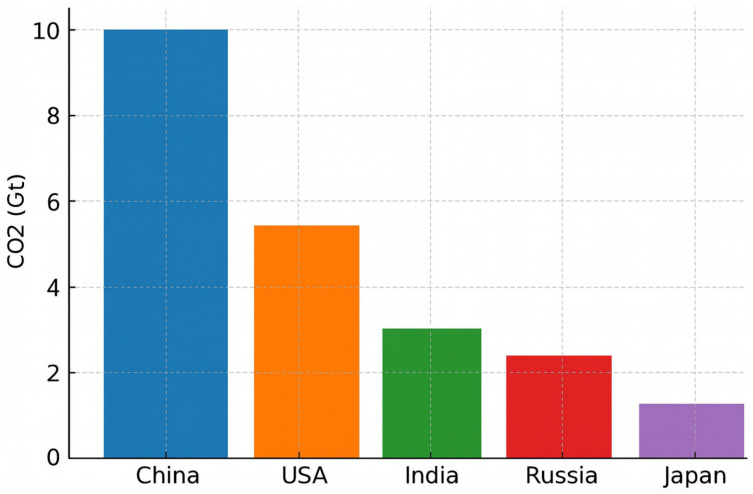
Example of incorrect use of colour in a bar graph. Each bar has a different, bright colour, not justified by the data. This makes it difficult to focus attention and increases the cognitive load required to distinguish between categories.

As shown in Figure 2, a bar chart displaying CO_2_ emissions by country (China, the USA, India, Russia, and Japan) assigns a distinct colour to each bar. While visually striking, this so-called “rainbow” design does not enhance interpretability; on the contrary, it introduces visual clutter. In the absence of a clear rationale for colour assignment, the viewer is left without perceptual guidance: no single bar visually dominates or stands out, and all elements compete equally for attention, generating perceptual noise and cognitive overload.

Furthermore, assigning a different colour to each bar implies the existence of distinct categorical meanings, which in this case are arbitrary and semantically unmotivated; the colours do not correspond to meaningful groupings such as continents or economic blocs. As a result, the colour encoding becomes superficial and distracting rather than informative.

From a cognitive standpoint, this design flaw is particularly relevant. Studies in cognitive psychology have established that working memory capacity is limited to approximately seven ± two items [8]. When each bar is assigned a unique colour, the viewer must mentally map and retain up to five colour-to-category associations, despite the lack of corresponding conceptual categories. This unnecessarily engages working memory resources and increases the cognitive cost of interpreting the visualisation.

Consequently, the user is forced to read and compare each bar individually, rather than gaining an immediate overview of the chart’s key message. The design not only slows down information retrieval but also obscures the intended emphasis, such as identifying the country with the highest emissions.

In contrast, applying colour selectively, using a neutral tone for all bars and a high-contrast hue only for the bar representing the highest value, enables targeted perceptual saliency and improves interpretive efficiency [8]. This approach capitalises on the principles of pre-attentive processing, minimising distraction and guiding the viewer’s gaze toward the most relevant data element.

**Figure 3:**
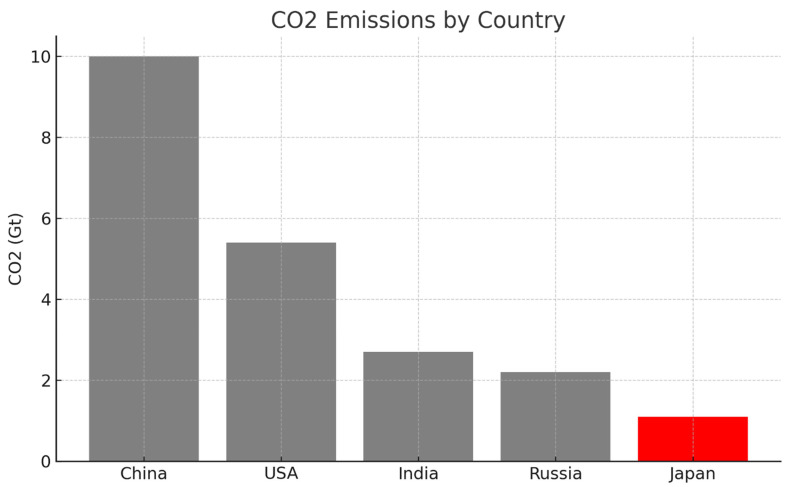
Example of the correct use of colour in a bar graph. All bars are grey except for the bar of interest (Japan), which is highlighted in red. This targeted use of the colour attribute immediately guides the eye to the key figure.

In Figure 3, the bar representing Japan, assumed here to be the data point of interest, either because it exhibits the lowest value or holds particular analytical significance, is coloured red, while the remaining bars are rendered in neutral grey. This configuration achieves a high visual contrast, immediately drawing the viewer’s attention to the red element through the mechanism of pre-attentive processing, as previously illustrated in Figure 1. Here, colour functions as an intentional and effective communication channel, allowing the viewer to instantly identify the key data point, Japan, without any need for legends or additional explanation.

This design aligns with dual-process theory: the observer is able to grasp the salient insight (e.g., that Japan has substantially lower CO_2_ emissions than other countries) at a glance via System 1, the intuitive and automatic cognitive mode. Only subsequently, using System 2, which governs deliberate, analytical reasoning, the user may examine exact values or compare across categories. What matters is that the primary message is successfully communicated before any conscious effort is required, thanks to a well-targeted use of a single pre-attentive attribute.

This principle is broadly generalisable. Minimising the use of heterogeneous visual encodings and instead applying one or very few pre-attentive features with a purpose enhances visual clarity and reduces cognitive burden. This holds true across visualisation formats:

In a scatter plot, a unique shape (e.g., a triangle) can highlight points belonging to a key category, while other points remain circles.

In an infographic, the use of a single tilted arrow among uniformly vertical arrows can signal an anomaly or deviation.

The overarching goal is to ensure that critical information is encoded by a unique and perceptually salient visual signal, processed effortlessly and unconsciously by the viewer.

Conversely, misuse of pre-attentive attributes occurs when no element is visually dominant or when too many elements simultaneously demand attention. A well-documented case in cognitive psychology involves conjunction search tasks. When a target differs from others only by a combination of two features (e.g., shape and colour), but not uniquely by either, it generally fails to trigger pre-attentive detection. For example, in a visual field containing blue circles and red squares, the target—a red circle—shares its colour with the squares and its shape with the blue circles. Lacking a distinctive single attribute, it fails to “pop out”, requiring a deliberate, point-by-point search via System 2.

In practical data visualisation, this is analogous to redundant or poorly structured encoding, where users must manually scan the graphic to determine what matters. The core principle remains: a pre-attentive attribute is effective only when it is uniquely assigned to the data element(s) intended to stand out. If everything is highlighted, nothing is.

The importance of these principles is particularly evident in clinical settings. In a study of anaesthesia monitors, researchers introduced a triangular indicator that changed size based on the probability of a change in patient vitals. This seemingly minor enhancement yielded significant benefits: physicians detected critical changes 14 s faster on average and missed fewer events (11% vs. 19%) compared to using a standard monitor without the pre-attentive cue [9,10,11]. This demonstrates how even subtle visual features, when designed to leverage pre-attentive processing, can markedly improve situational awareness and response time.

**Figure 4:**
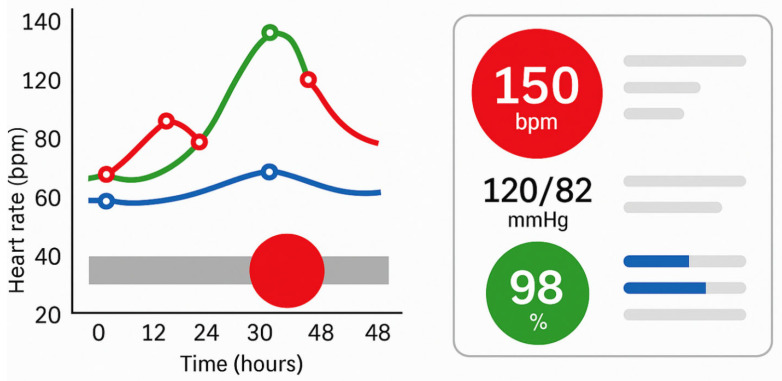
Example of pre-attentive visual attributes in clinical dashboards. **Left panel**: line chart showing physiological trends over time; coloured markers indicate critical changes, with a large red circle highlighting a major event. **Right panel**: icon-based vitals display using colour and size to convey urgency (e.g., red = alarm, green = stable), enhancing rapid interpretation.

Similarly, Pollack et al. compared different ways of presenting electronic health record (EHR) data to help physicians prioritise inpatients. Visualisations that employed colour or spatial encoding to emphasise clinical severity significantly reduced cognitive load during the prioritisation process [3]. In other words, when relevant patterns and outliers are visually foregrounded, clinicians can process critical information more quickly and with less effort. The images panel in Figure 4 illustrates how targeted use of pre-attentive attributes, such as colour, size, and shape, can enhance the effectiveness of clinical visualisations by directing attention to critical data within milliseconds. The left panel simulates a temporal patient monitoring chart, where visual saliency (red highlight) enables rapid detection of anomalies without detailed inspection. The right panel represents a simplified clinical dashboard that uses colour-coded icons and minimalist layouts to reduce cognitive load and enable intuitive interpretation. This approach aligns with best practices for real-time decision support in high-stakes environments like intensive care or oncology.

Additional research on diagnostic decision-making has shown that well-designed, intuitive graphical interfaces in EHR systems can encourage System 1 reasoning, based on pattern recognition, even among novice physicians. As demonstrated in a study on diagnostic training [12], such interfaces improved decision speed without compromising accuracy, suggesting that intuitive visual design enhances both efficiency and safety in high-stakes environments.

Despite the potential of visualisation to improve understanding, inadequate design can impair or distort perception. One of the most pervasive errors is the use of the rainbow colour scale, which, while visually appealing, is widely recognised as ineffective for quantitative interpretation. Rainbow scales introduce non-linear perceptual gradients, create false discontinuities, and are inaccessible to individuals with colour vision deficiencies. In epidemiological mapping, their use can obscure genuine trends or highlight non-existent contrasts, misguiding interpretation.

Other typical errors include the following:

- Overloading the graph with data series, unnecessary labels, or cluttered formats (e.g., 3D pie charts), which overwhelms the viewer;
- Axis manipulation, such as truncating the Y-axis, which can exaggerate visual differences between data points.

These issues were widely observed during public health communication in the COVID-19 pandemic, where misleading graphs, such as logs without explanation or dashboards showing only instantaneous values, led to confusion among both experts and the general public. This underscores the need for ethically responsible visualisation, especially when informing policy or public behaviour.

## 3. Ethical Considerations in Medical Data Visualisation

In the healthcare context, where data visualisation can influence clinical decisions, public policies, and individual behaviours, ethical responsibility in design is crucial. Poorly constructed visualisations can not only mislead but also exacerbate inequalities, propagate bias, or induce excessive anxiety in patients. It is therefore essential to integrate ethical principles into the design, implementation, and dissemination of medical data visualisations.

Visualisations must present data truthfully and without distortion. Common errors such as truncated axes, deceptive colour gradients, or the improper use of 3D effects can exaggerate or mask differences. These choices, whether intentional or not, risk misleading clinicians or the public, especially when used in epidemiological dashboards or healthcare communication. Ethical visualisation requires fidelity to the underlying data and transparency in design choices.

Medical visualisations often derive from sensitive personal data. Ethical practice requires strict compliance with privacy regulations (e.g., GDPR, HIPAA), especially in dashboards that allow for in-depth analysis of individual patients. Designers must ensure that identifiers are anonymised and access control mechanisms are implemented. When developing interactive or AI-based dashboards, data provenance and ownership must be respected, ensuring that patients and communities retain control over their data.

Patient-facing images must take into account psychological and emotional effects. Dashboards that show prognosis, risk of relapse, or treatment failure should be designed to inform, not alarm. For example, the use of strong colours (such as red) or alarming icons can increase anxiety, especially in oncology. Ethical visualisation promotes clarity and support without sensationalism, ideally co-designed with patients to meet their information needs and literacy levels.

Visual representations often reflect biases inherent in the data collection and modelling processes. For example, predictive dashboards based on unrepresentative training cohorts can perpetuate racial, gender, or socioeconomic bias. Ethical design involves clearly communicating uncertainty, documenting data limitations, and avoiding false precision. Visualisations should include contextual annotations when data quality, completeness, or generalisability are limited.

Visualisations should be accessible to diverse users, including people with visual impairments, low health literacy, or limited access to technology. Ethical design includes the use of perceptually consistent palettes (e.g., cividis, viridis), appropriate font sizes, and responsive layouts. Multilingual support and alternative text descriptions can further reduce inequities in access to information.

Although there is no universal regulatory standard for medical visualisation, ethical frameworks can be derived from related fields. For example, the Fair Information Principles (FIPP) [9] and the European Commission’s Ethical Guidelines for Trustworthy Artificial Intelligence [10] offer relevant principles such as transparency, accountability, and non-maleficence. Furthermore, the emerging concept of visualisation ethics encourages explicit documentation of design intent, audience, and potential risks. In short, ethical visualisation in medicine goes beyond aesthetics and usability: it safeguards trust, fairness, and patient safety. As digital tools become increasingly central to clinical practice and public communication, integrating ethical considerations into every stage of the visualisation lifecycle, from data curation to user testing, becomes not only optional but essential.

## 4. Cognitive Limitations of the User and the Role of the Two Thinking Systems

In addition to leveraging pre-attentive mechanisms, the design of effective data visualisations must take into account the cognitive limitations of users, particularly those related to working memory and the dual-process model of cognition, as articulated by Kahneman’s System 1 and System 2 framework [13,14,15].

Working memory, or short-term memory, is the cognitive workspace where information is temporarily held and actively processed during reasoning tasks. It is, however, notoriously limited in capacity. While the classic estimate by Miller suggested a span of seven ± two items, more recent research suggests that the true capacity may be closer to four items, especially in the absence of chunking strategies.

This limitation has direct implications for visualisation design. Overly complex graphics, for example, charts with ten distinct colour-coded categories, can overwhelm the user’s working memory, making it difficult to retain and interpret information across multiple fixations. Similarly, if a comparison requires mentally tracking the outcome of previous comparisons (e.g., evaluating eight bars in a chart one by one), cognitive load increases significantly.

To mitigate this, effective visualisations should aim to achieve the following:

- Minimise memory load by reducing the number of simultaneously encoded variables;
- Use direct labelling instead of legends, so the viewer does not have to refer back and forth;
- Segment complex information across multiple simpler graphs, if necessary.

### Kahneman’s Dual-System Theory in Visual Interpretation

**Figure 5:**
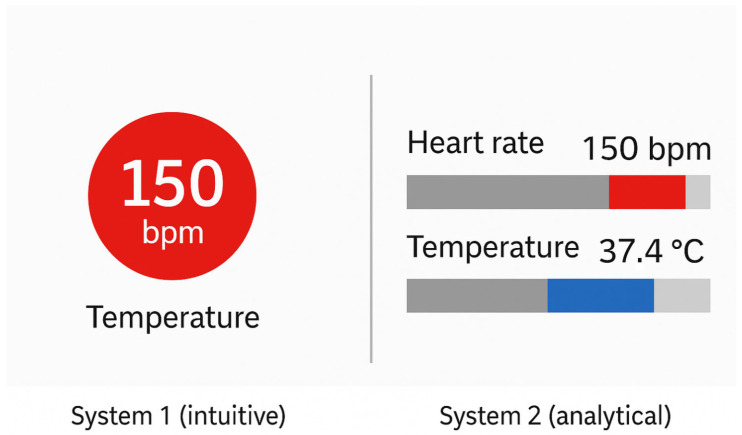
Cognitive systems in visualisation. **Left**: effective design supports fast, intuitive recognition (System 1) through salient visual cues. **Right**: poor design forces slow, effortful analysis (System 2) due to a lack of visual clarity.

According to Kahneman’s dual-process model, human cognition operates via two interacting systems [13] (Figure 5):

- System 1: Fast, intuitive, and automatic. It requires minimal effort and operates unconsciously. This system governs initial, low-effort perception, such as the immediate recognition of a salient data point via a pre-attentive attribute.
- System 2: Slow, deliberate, and analytical. It requires focused attention and is responsible for logical reasoning and complex decision-making. It becomes active when tasks require interpretation, calculation, or comparison of precise values.

In the context of data visualisation, both systems ideally operate in sequence. A well-designed visualisation allows System 1 to quickly provide a meaningful overview: highlighting the most important feature (e.g., the highest value, an outlier, or a trend direction). Subsequently, System 2 enables deeper analysis—reading exact numbers, comparing between groups, or consulting legends.

When this transition is seamless, cognitive effort is minimised, and comprehension is efficient. Conversely, in poorly designed visuals, System 1 may produce misleading impressions due to excessive or ambiguous stimuli (e.g., inconsistent use of colour or shape). In such cases, System 2 must intervene to “repair” the initial impression through detailed, resource-intensive analysis.

For instance, the following apply:

- If two unrelated data points are inadvertently assigned the same colour, System 1 may infer a non-existent association.
- If no element stands out due to visual homogeneity, the user must scan the entire graphic manually to locate relevant information.

Such designs place the burden of interpretation on System 2, contradicting the goal of reducing cognitive effort.

Importantly, System 1 is not immune to perceptual biases, particularly when interpreting visual encodings that involve area, angle, or curvature. Human perception is naturally better at judging position and length on a common scale than comparing angles (as in pie charts) or surface areas (as in bubble charts). This has been well documented since the foundational work of Cleveland and McGill [15], who ranked visual encoding types by perceptual accuracy. Their hierarchy indicates the following:

- Position and aligned length are the most accurate and cognitively efficient;
- Area, angle, and colour (for quantitative data) are more error-prone.

Thus, for tasks that require precise quantitative comparison, bar charts or dot plots are generally superior to pie charts or bubble charts, as they allow System 1 to work more effectively, producing accurate judgements with minimal effort and reducing reliance on System 2.

Beyond expert users, patients and citizens also engage with medical visualisations, especially in telemedicine platforms, patient dashboards, and public health communication. These users often have limited visual literacy or domain knowledge, making them more vulnerable to confusing or overloaded visual designs.

Effective communication in this context requires the following:

- Minimalist, intuitive graphics, free from unnecessary technical complexity;
- Use of icons, clear labels, and plain language to aid comprehension;
- Focusing on essential information only, avoiding dense legends or fine-grained colour keys.

In summary, designing visualisations with cognitive constraints in mind means aiming for perceptual accessibility: ensuring that critical information is visually evident and understandable with as little mental effort as possible. When System 1 is effectively guided by perceptual cues, and System 2 is reserved for meaningful deeper analysis, visualisations become not only more usable but also more trustworthy and cognitively efficient.

In addition, two effective strategies to reduce cognitive load are chunking and progressive disclosure.

Chunking: This technique involves grouping related pieces of information into cohesive units or “chunks” that are easier to process. For example, instead of displaying 12 lab values individually, results can be clustered into meaningful groups (e.g., liver function, renal profile, inflammatory markers) with summarised indicators. Visual grouping can be reinforced using spatial proximity, background shading, or enclosing boxes—leveraging Gestalt principles to support perception and memory. This approach aligns with Miller’s foundational insight that working memory can hold approximately seven ± two items [12], and more recent evidence suggesting a limit of around four ungrouped elements.

- Progressive Disclosure: This principle involves initially showing only high-level or essential information and allowing the user to drill down for further detail if needed. For instance, a dashboard may initially display only abnormal or trending values, with collapsible panels to reveal the full history or numerical tables upon user interaction. This limits the visual complexity at first glance and supports System 1-driven attention while still preserving access to data for System 2-based analysis. Interactive visualisations that allow for on-demand exploration, such as timelines where treatment details appear on hover or click, exemplify this principle.

When used together, chunking and progressive disclosure allow for presenting complex information in a cognitively ergonomic manner. These strategies reduce the need for constant mental comparison or memory retention and align the presentation of data with the natural flow of clinical reasoning. Incorporating them not only enhances usability but also supports faster, safer clinical decision-making under conditions of cognitive load and time pressure.

## 5. Design Recommendations for Effective Health Visualisations

Based on the perceptual and cognitive principles discussed, the following guidelines can support the development of clinically effective dashboards, telemedicine interfaces, and public health reports. These best practices aim to enhance visual clarity, minimise cognitive overload, and ensure that critical information is conveyed rapidly and intuitively [16].

1. Leverage pre-attentive attributes purposefully.

Highlight critical elements using high-contrast colours (e.g., abnormal values in red), employ distinctive shapes or icons to categorise different information types (e.g., heart vs. lung parameters), and use size or thickness to emphasise high-priority data (e.g., central values in dashboards). The aim is to guide System 1 processing and reduce interpretive effort.

2. Avoid excessive visual encoding.

Resist the temptation to combine multiple attributes—colour, shape, pattern, size—simultaneously, especially when dealing with more than 4–5 categories. Overloading visual channels leads to confusion and working memory saturation. Instead, focus on a few salient, semantically meaningful visual cues.

3. Use perceptually robust colour scales.

Select perceptually uniform palettes (e.g., viridis, cividis) that maintain legibility across different visual conditions and for users with colour vision deficiencies. Avoid problematic combinations (e.g., red/green); when colour alone is insufficient, reinforce meaning with redundant encodings (e.g., adding icons or labels).

4. Apply Gestalt principles and visual hierarchy.

Structure information using grouping, alignment, and proximity. Related elements (e.g., all vital signs) should be visually grouped; unrelated sections should be clearly separated. Place critical content in visually dominant positions (top-left or centre) and consider size, brightness, or position to establish a clear visual hierarchy.

5. Progressively reveal complexity.

For data-dense environments, such as ICU dashboards or complex reports, adopt a progressive disclosure strategy: show a concise overview first, with options to drill down into specific sections. This reduces initial cognitive load while maintaining access to detailed insights.

6. Ensure clear and context-aware labelling.

Each visual element should be accompanied by descriptive titles, labelled axes, and interpretable legends. However, well-designed visuals can reduce dependence on legends: for example, if red universally signals alarm, repetition may be unnecessary. Clarity must be maintained without redundancy.

7. Adapt design to usage context.

Tailor visual features to the medium and environment. For example, dark backgrounds and high-contrast colours are effective for ICU monitors in low-light conditions; light, low-saturation tones may be ineffective in print. Consider responsiveness, font size, and avoidance of clutter in screen-based tools.

8. Adopt a user-centred design process.

Involve target users—clinicians, nurses, patients—throughout the design cycle. Participatory design and usability testing can reveal unanticipated issues, such as unclear iconography or missing data. In infection control dashboards, for instance, collaborative design has been shown to improve clinical utility.

9. Minimise extraneous cognitive load.

Following the principles emphasised by authors such as Edward Tufte [17], all elements in a graphic should serve a clear purpose. Avoid non-informative “chartjunk” such as 3D effects, decorative icons, or excessive gridlines. Simplicity enhances clarity and user focus.

10. Validate communicative effectiveness empirically.
11. Before deployment, evaluate the visualisation with real users in realistic or simulated scenarios. Assess metrics such as the following:
  ○ Time required to locate key information;
  ○ Accuracy in interpreting trends or outliers;
  ○ Subjective ratings of clarity and usability.

Such testing enables iterative refinement and ensures that the visualisation truly supports clinical insight and decision-making.

## 6. Applied Visualisation in Oncology: Supporting Precision Medicine Through Graphical Intelligence

In the era of precision oncology and big data, data visualisation has emerged as an indispensable tool for supporting clinicians and researchers in the interpretation of multidimensional and heterogeneous data. As cancer care increasingly relies on molecular profiling, real-time monitoring, and patient-specific treatment pathways, the ability to translate complex data into intuitive visual representations is essential for evidence-based and timely clinical decision-making.

This section presents concrete examples, drawn from recent scientific literature, illustrating how various visualisation techniques are currently employed in oncology to guide individualised treatment decisions; enable risk stratification and prognosis; support longitudinal monitoring of disease and therapeutic response; integrate clinical, genomic, and imaging data across disparate health information systems; and provide clinicians with interactive, user-friendly dashboards for managing complex patient profiles.

For each example, we describe the most appropriate visualisation modality, such as genomic heatmaps, prognostic nomograms, temporal event timelines, and multilayered interactive dashboards, along with key references from the scientific literature that demonstrate their effectiveness in practice.

### 6.1. Visualising Genomic Complexity in Precision Oncology: Tools for Molecular Tumour Boards

The implementation of targeted therapies in oncology requires the integration and synthesis of diverse data streams, including genomic, molecular, and clinical information, to identify actionable, patient-specific therapeutic targets [18,19,20,21,22,23,24,25,26]. In this context, Molecular Tumour Boards (MTBs) play a central role in evaluating complex cases and proposing personalised treatment strategies based on the molecular profile of individual tumours.

To support this process, dedicated visual platforms for genomic data interpretation have been developed. One of the most widely adopted tools is the cBioPortal for Cancer Genomics, an open-source web application designed to facilitate the visual exploration of next-generation sequencing (NGS) data. cBioPortal enables the interactive and intuitive representation of complex genomic profiles, streamlining the preparation of clinical case discussions and enhancing multidisciplinary communication during MTB sessions.

**Figure 6:**
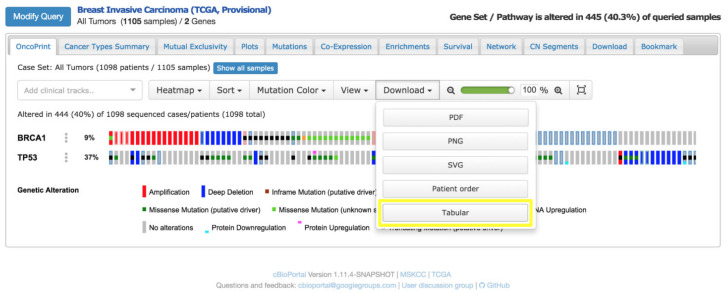
OncoPrint visualisation from cBioPortal showing genomic alterations in BRCA1 and TP53 genes across breast cancer samples (TCGA-BRCA cohort).

A notable application of cBioPortal is the OncoPrint, a compact heatmap-like visualisation tailored to summarise genetic alterations across patient cohorts [27]. In an OncoPrint [28] (Figure 6), the following apply:

- Rows correspond to genes of clinical relevance (e.g., tumour suppressors, oncogenes),
- Columns represent patient samples,
- Coloured cells encode the type of genomic alteration (e.g., mutation, copy number amplification, deletion, structural rearrangement).

This visual layout allows clinicians to quickly identify patterns of co-occurrence or mutual exclusivity among mutations and relate them to therapeutic opportunities, such as eligibility for targeted therapies or clinical trials.

Beyond OncoPrints, another commonly used visualisation in precision oncology is the lollipop plot (or candy diagram), which displays mutations along the linear structure of a protein. These charts show the following:

- The functional domains of the protein,
- The position and frequency of individual mutations (illustrated as lollipops along a horizontal axis).

**Figure 7:**
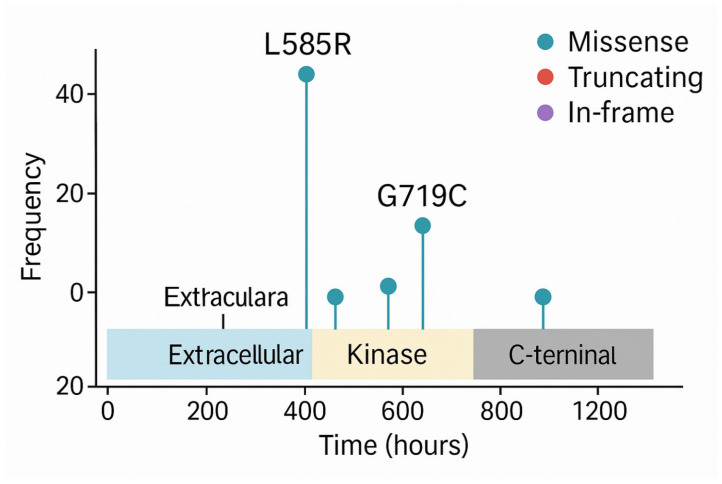
Lollipop plot of EGFR gene mutations. The horizontal axis represents the linear structure of the EGFR protein, including its key functional domains (e.g., kinase domain). Each “lollipop” marker indicates the position and frequency of a specific mutation: the stem corresponds to the amino acid position, the circle size indicates mutation frequency, and the colour may represent mutation type.

Lollipop plots (Figure 7) are particularly useful for detecting hotspots, clusters of mutations in functionally critical regions, such as kinase domains, which may predict sensitivity to specific inhibitors. For example, recurring mutations in the kinase domain of EGFR are indicative of likely responsiveness to tyrosine kinase inhibitors (TKIs).

In a German case study, cBioPortal [29] was successfully integrated into hospital information systems, enabling direct access to EHRs and laboratory databases, effectively transforming the platform into a Clinical Decision Support System (CDSS) for precision oncology. This integration facilitated seamless access to genomic data within the clinical workflow, enhancing the speed and precision of therapeutic recommendations.

These forms of visual representation, OncoPrints, lollipop charts, and others, serve not merely as aesthetic aids but as critical cognitive tools for linking molecular alterations with clinical actionability, bridging the gap between bioinformatics data and therapeutic decision-making in modern oncology.

Despite the growing use of visual analytics platforms in Molecular Tumour Boards, several barriers hinder their seamless integration into clinical workflows. A major challenge is interoperability: genomic platforms often lack direct interfaces with hospital electronic EHRs, requiring manual data import/export or custom API development. Additionally, clinicians may face a steep learning curve when using tools originally designed for researchers rather than healthcare providers.

To overcome these challenges, adopting a modular design can be effective, allowing selective integration of components (e.g., OncoPrints, mutation diagrams) into existing dashboards. Additionally, integrating usability testing protocols in the implementation phase can ensure that the interface meets the specific needs and cognitive expectations of clinical users. This includes involving end users (oncologists, pathologists, nurses) in iterative testing and adapting functionality to different skill levels.

Educational initiatives and institutional support are also essential to promote adoption. Studies suggest that with proper onboarding and workflow integration, platforms like cBioPortal can evolve from research tools to powerful CDSS integrated into daily practice.

### 6.2. Visual Tools for Prognostic Stratification in Oncology

Prognostic stratification plays a pivotal role in oncology, enabling clinicians to adapt the intensity and type of care to the individual patient’s risk of recurrence, metastasis, or treatment-related complications. While statistical models can accurately estimate prognosis using multiple clinical and molecular variables, their outputs are often presented in numerical form, making them less accessible in routine clinical decision-making. Graphical representations of prognostic models significantly enhance their usability, interpretability, and bedside applicability.

One of the most established tools in this domain is the oncology nomogram, a graphical device that translates multivariable regression models into an intuitive visual interface. These diagrams allow clinicians to input patient-specific variables—such as age, tumour stage, histological grade, and biomarker status—and obtain a corresponding predicted probability of a clinical outcome (e.g., 5-year survival, disease recurrence, or risk of metastasis). Each variable is mapped onto a point scale, and the total cumulative score correlates with the probability of the outcome. In this way, the nomogram provides a quantitative yet personalised assessment of prognosis.

Numerous studies have validated the clinical utility and predictive accuracy of well-constructed nomograms [30,31,32]. These evaluations typically involved retrospective or prospective cohort analyses where nomogram predictions were compared against actual clinical outcomes. Predictive accuracy was quantified using discrimination metrics such as the concordance index (C-index) or area under the ROC curve (AUC), while calibration plots assessed the agreement between predicted and observed outcomes. Clinical utility was often examined through decision curve analysis (DCA), which estimates the net benefit of using the nomogram across a range of threshold probabilities, thereby demonstrating its practical value in guiding therapeutic choices.

By visualising the relative impact of each prognostic factor, these tools assist both clinicians and patients in understanding individualised risk profiles, supplementing traditional staging systems such as TNM, and supporting more nuanced treatment planning. For instance, a nomogram may indicate that a patient with advanced age, high tumour stage, and unfavourable biomarker expression has a low likelihood of benefit from standard therapies, prompting consideration of more aggressive interventions or enrolment in clinical trials.

Another widely used visualisation for prognostic stratification is the Kaplan–Meier (KM) survival curve, which compares survival distributions across risk groups. KM survival curves are non-parametric estimators used to assess the probability of survival over time, particularly useful when data are censored. The method calculates the survival function representing the probability that a subject survives beyond time; at each event time, the KM estimator updates the survival probability by multiplying the prior survival probability by the conditional survival probability at that time point. Censoring, cases where a subject leaves the study or is event-free at the end, does not affect earlier survival estimates, making KM curves especially suitable for clinical studies where not all patients experience the event of interest during follow-up. Statistical comparisons between survival curves (e.g., low-risk vs. high-risk groups) are often performed using the log-rank test to evaluate differences in survival distributions. KM curves are commonly employed to illustrate the prognostic separation achieved by a given stratification method, whether based on clinical scores, molecular signatures, or composite indices. For example, a KM plot might demonstrate a statistically significant difference in survival between low-risk and high-risk cohorts, thus justifying differentiated therapeutic strategies.

In the context of molecular oncology, heatmaps offer another powerful visual modality for stratification. Gene expression heatmaps cluster tumour samples based on transcriptomic profiles, often revealing molecular subtypes with distinct prognostic trajectories—as exemplified in breast cancer by the Luminal A/B, HER2-enriched, and Basal-like classifications. The use of colours and hierarchical clustering allows for rapid identification of multi-gene expression signatures associated with favourable or poor outcomes. These patterns not only inform prognostic stratification but also influence decisions regarding adjuvant therapy intensification or de-escalation.

In all these cases, data visualisation bridges the gap between statistical modelling and clinical actionability. By translating numerical risk estimates and model coefficients into immediate, interpretable visuals, these tools facilitate shared decision-making, support communication within the clinical team, and enable more precise, personalised care pathways.

### 6.3. Temporal Visualisation in Oncology: Tracking Treatment Trajectories and Tumour Evolution

Temporal data visualisation is especially valuable in oncology [33,34,35,36,37,38,39,40,41,42,43,44,45,46,47], where monitoring both tumour progression and treatment administration over time is crucial for clinical decision-making. Translating a patient’s clinical course into a visual timeline or interactive diagram facilitates a clearer understanding of therapeutic sequences, responses, and disease dynamics—for both clinicians and patients.

A growing body of literature supports the effectiveness of such representations. For instance, a recent study investigated the use of icon-based treatment timelines to help patients in oncohematology settings understand and recall their care trajectory. The results were striking: over 82% of patients correctly recalled key treatment phases, and the visual timelines were also positively evaluated by physicians [40]. These findings underline the role of well-designed temporal visualisations in enhancing patient engagement, memory retention, and clinical communication.

From a clinical standpoint, timelines can clearly mark the following:

- Cycles of therapy and surgical interventions;
- Periods of remission or stable disease;
- Follow-up appointments and disease relapse.

Having a graphical summary of the treatment history available during patient consultations can support more precise planning (e.g., assessing the interval since the last dose or scan) and foster shared decision-making.

**Figure 8:**
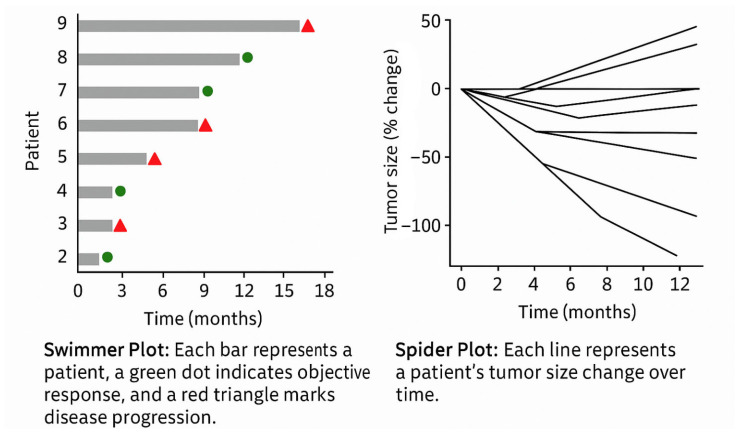
Swimmer and spider plots.

In the context of clinical trials and longitudinal studies, temporal visualisation is also central to monitoring therapeutic outcomes. Two widely adopted formats include the following (Figure 8):

- Swimmer Plots: Each patient is represented as a horizontal bar along a time axis, indicating duration on treatment, response onset, progression, and treatment discontinuation. These plots offer a compact summary of individual trajectories across a cohort, enabling immediate pattern recognition (e.g., delayed vs. early responders).
- Spider Plots: These show the evolution of tumour size over time for multiple patients. The y-axis represents absolute or percentage change from baseline, while the x-axis indicates timepoints. Each line represents a patient’s tumour burden trend, enabling quick identification of responders, non-responders, and patterns of relapse or progression. The “web-like” layout makes it easy to detect heterogeneity in response across a population.

Such visualisations are pivotal in assessing the efficacy and durability of treatment responses and can be adapted to individual patient care. For example, in routine practice, line graphs are commonly used to plot the following:

- Tumour marker levels (e.g., PSA in prostate cancer, CA-125 in ovarian cancer);
- Tumour diameters across imaging follow-ups, often normalised to baseline, to track objective response to therapy.

These charts provide clinicians with an at-a-glance overview of therapeutic trends, supporting the timely detection of disease progression or treatment failure.

Recent advances in medical imaging and artificial intelligence have further expanded the potential of temporal visualisation in oncology. Innovative tools—such as the LesionViewer prototype and radiomics platforms—offer the ability to generate synchronised visual timelines of tumour response using CT or MRI imaging data.

These systems can achieve the following:

- Automatically segment tumours and surrounding organs;
- Track volumetric changes over time;
- Visually highlight lesions using colour-coded overlays to indicate size reduction or progression;
- Generate quantitative plots of tumour volume across treatments.

For example, a liver metastasis can be visualised on successive CT scans with contour overlays, showing shrinkage or growth over time. This facilitates longitudinal assessment that complements traditional radiology reports.

According to Chang et al. (2025) [41], integrating automated lesion tracking algorithms into clinical workflows allows real-time visual assessment of how tumours are responding to each line of therapy. These tools enhance sensitivity in detecting subtle but clinically meaningful changes, thereby enabling earlier intervention and more informed modifications of treatment regimens.

While further large-scale validation is needed, these technologies point toward a future in which dynamic, radiologically anchored dashboards become routine in oncology practice—augmenting human interpretation with machine-assisted visual insights.

### 6.4. Multilevel Dashboards and Integrated Visualisation in Oncology: Enabling Data-Driven Precision Care

In modern oncology, interactive dashboards are emerging as essential tools for aggregating and visualising multisource clinical data in real time. When thoughtfully designed, these dashboards provide clinicians with a comprehensive overview of patient status while enabling seamless access to granular data through multilevel navigation interfaces. Such systems embody the principles of hierarchical visualisation, allowing users to transition fluidly from population-level insights to patient-specific details.

A notable implementation of this approach is described in the work by Seol et al. [42], which details a two-tier dashboard system for managing patients with colorectal cancer. At the cohort level, the dashboard displays aggregate metrics such as patient demographics, incidence and mortality trends, treatment distributions by TNM stage, and therapeutic flow diagrams. These views assist clinicians and administrators in identifying epidemiological patterns and evaluating the consistency of treatment pathways. At the patient level, the dashboard presents a chronological, icon-enhanced timeline of the individual’s clinical history—including laboratory tests, imaging studies, therapies, and interventions—often augmented with trend graphs for lab values and time bars for treatments. This temporal visualisation enables rapid comprehension of disease evolution, therapeutic responses, and follow-up needs, all within a unified interface.

A major strength of these dashboards lies in their interactivity and customisability. Users can complete the following:

- Filter cohorts by biomarkers or stage and observe real-time updates of associated survival curves or treatment trends;
- Toggle data views for individual patients to display specific clinical parameters, visual summaries, or progress notes.

A 2025 study assessing the adoption of such dashboards in a large oncology centre reported high satisfaction among physicians (scores > 4/5), highlighting their practical utility and workflow integration. For example, during follow-up visits, clinicians may review dashboard summaries beforehand and update key parameters during the consultation, with all changes reflected instantaneously, enhancing both efficiency and data accuracy.

In the context of multidisciplinary tumour boards (MTBs), these dashboards function as visual cockpits, displaying tumour genomic profiles (e.g., OncoPrints or annotated mutation tables); time-series plots of tumour markers; key radiological images with measured lesions; and embedded links to clinical guidelines and trials.

This integrated visual interface allows the care team to evaluate multiple dimensions of a case simultaneously and reach evidence-based consensus decisions. The ability to interact with the data—zooming, filtering, annotating—transforms clinicians from passive recipients of reports into active interpreters of complex datasets.

True data-driven care requires robust integration across disparate clinical systems: EHRs, genomic platforms, diagnostic imaging systems (PACS), pharmacy and treatment records, tumour registries, and more. A state-of-the-art example is the Yonsei Cancer Data Library (YCDL), a multimodal data integration infrastructure implemented at an academic cancer centre in South Korea.

The YCDL integrates data from over 170,000 cancer patients, spanning 11 tumour types and encompassing more than 800 clinical, genomic, pathological, and imaging variables per patient. Through ETL (Extract–Transform–Load) processes and natural language processing (NLP) for free-text data, the system maintains high-quality, structured records with >92% logical accuracy. On this foundation, an advanced CDSS dashboard was developed, capable of visually rendering the full clinical trajectory—from diagnosis through treatment, remission, relapse, and end-of-life care.

Among its features, the YCDL-based platform allows for real-time survival stratification based on integrated variables; interactive exploration of longitudinal patterns (e.g., biomarker evolution and treatment response); automated identification of patients eligible for clinical trials; and hypothesis generation for research (e.g., drug–biomarker correlations).

The availability of a single-point visual interface, rather than requiring separate logins to EHRs, genomic tools, and imaging viewers, significantly reduces cognitive load, enhances diagnostic confidence, and mitigates clinician fatigue.

Although building such systems involves considerable technical and organisational complexity, including adherence to interoperability standards like the Observational Medical Outcomes Partnership Common Data Model, robust data governance, and privacy protections, the benefits outweigh the investment. Once implemented, these platforms unlock possibilities for predictive analytics, such as risk stratification or adverse event prediction; decision automation, including clinical alerts and drug-matching engines; and clinical trial optimisation, through real-time patient screening.

Notably, commercial solutions such as NAVIFY Oncology Hub, Syapse, and Flatiron Assist are actively being evaluated for integration with hospital EHRs. These platforms aim to provide oncology care teams with synoptic dashboards embedded within daily workflows, offering consolidated views of demographics, comorbidities, genomics, treatment paths, and clinical guidance, available at the point of care.

The transition from research infrastructure to mainstream practice reflects a broader shift toward visual, integrated, and intelligent oncology, where complex data is not just stored but actively used to improve care, personalise therapy, and support real-time clinical decisions.

### 6.5. Applications in Bioengineering and Oncological Data Visualisation

In oncology and biomedical engineering, the effective visualisation of complex and multivariate data is not merely an aesthetic concern but a functional necessity (Table 2). The use of pre-attentive visual attributes can drastically reduce the cognitive load required to interpret high-dimensional data, enabling faster and more accurate clinical and research decisions.

In radiomics and quantitative imaging, for example, tumour features extracted from Computed Tomography or Magnetic Resonance images (e.g., heterogeneity, edge sharpness, radiodensity patterns) are increasingly visualised through colour-encoded overlays directly on the medical images [48,49,50]. These overlays use perceptually salient colour gradients to indicate severity or statistical deviation from normative models, allowing radiologists to spot areas of interest pre-attentively, within milliseconds, before focused analysis. Studies have shown that when uncertainty in AI-generated segmentations is encoded via subtle but distinct visual cues (e.g., fuzzy borders or transparency), clinicians make more accurate judgments and are better able to trust or challenge algorithmic suggestions.

In bioengineering contexts, such as tumour-on-chip systems or 3D bioprinted tissue models, real-time biosensor data (oxygen, pH, mechanical strain, temperature) are often monitored via interactive dashboards. These dashboards rely heavily on intuitive graphical metaphors; for example, a circular gauge turning from green to red as oxygen tension drops or microfluidic flow diagrams using animated vectors to show flow irregularities. Pre-attentive signals like motion direction or colour change are essential to detect system failure or critical deviations in vitro before downstream decisions (e.g., drug administration timing). Similarly, shape changes (e.g., expanding bubbles or warning triangles) can indicate physical deformations or instability in tissue scaffolds.

Moreover, in patient-facing tools, such as mobile apps for monitoring symptoms during chemotherapy, pre-attentive design becomes a matter of usability and equity. Patients with no medical background benefit from dashboards that use universally interpretable icons, traffic-light colour codes, and progressive disclosure of information. A red icon with an exclamation mark next to a fatigue rating immediately signals that something is abnormal, without requiring detailed interpretation.

From a design perspective, integrating biomedical ontologies and semantic layers into visual interfaces, such as linking gene expression heatmaps with pathway diagrams, offers a layered understanding where biological function is visually connected to data patterns. Here, perceptual grouping and spatial proximity principles (from Gestalt psychology) can be leveraged to show which genes act together or diverge across tumour subtypes.

In short, the convergence of perceptual psychology, oncology, and bioengineering requires an interdisciplinary visual language that can support both high-level pattern recognition and fine-grained analytical scrutiny. Applying these principles systematically improves clarity, reduces error, and enhances the decision-making capabilities of both clinicians and biomedical researchers.

To strengthen the reliability and reproducibility of oncological data visualisations, it is essential to incorporate quantitative assessments that evaluate the effectiveness of pre-attentive attributes (such as colour, shape, orientation, and size). These attributes guide user attention before conscious processing and play a critical role in clinical interpretation tasks.

Several quantitative methods and usability metrics can be employed, including the following:

- Visual search task performance: Measuring accuracy and reaction times in target detection tasks across varying pre-attentive feature designs.
- Eye-tracking studies: Used to determine gaze fixation, time to first fixation, and scan paths when interpreting radiological maps or radiomic outputs.
- Signal detection theory (SDT) metrics: Such as sensitivity and criterion, which quantify the observer’s ability to discriminate between classes under different visual encoding schemes.
- Cognitive load measures: Using NASA-TLX (Task Load Index) [51] or similar tools to assess mental effort induced by different visual styles.
- User preference surveys and SUS (System Usability Scale) scoring [52]: To supplement performance-based measures with subjective evaluations.

In the field of radiology and oncological imaging, emerging standards for evaluating visual data interfaces include the following:

- RSNA’s Structured Reporting Templates, which promote consistency in layout and highlight the importance of visual hierarchy and clarity [53].
- The Image Biomarker Standardisation Initiative (IBSI), which, although focused on radiomic feature extraction, provides foundational standards for the reproducibility of image-based quantitative results [54,55,56,57].
- ISO 9241-210 [58] and IEC 62366 [59] standards on human-centred design and usability engineering for medical devices, offering broader frameworks applicable to visualisation tools.

Incorporating these frameworks and performance metrics would facilitate objective validation of the visualisation strategies used in radiomics and radiogenomic research. Future work may also consider benchmarking visual encoding techniques across standardised datasets to optimise interpretation efficiency and reduce diagnostic variability.

## 7. Conclusions

An in-depth understanding of visual perception principles and the strategic use of pre-attentive attributes are critical determinants of success in the design of effective data visualisations in healthcare. Medical data is inherently complex, multidimensional, and often voluminous; only through deliberate and evidence-informed design can such data be rendered immediately comprehensible, usable, and clinically actionable.

Numerous case studies and empirical research underscore that well-constructed visualisations can achieve the following:

- Reduce the cognitive load on clinicians;
- Accelerate the detection of critical events;
- Enhance the accuracy and timeliness of clinical decision-making [3].

Conversely, poor design choices—such as ambiguous visual encodings, inappropriate colour schemes, or lack of visual hierarchy—may lead to misinterpretations or even unsafe clinical decisions. In the healthcare domain, where decisions often carry life-altering consequences, data visualisation is not merely an issue of aesthetics or convenience; it is a matter of clinical safety, efficiency, and communication integrity.

Therefore, it is strongly recommended that healthcare organisations and system developers conduct the following:

- Invest in training and awareness on visual communication principles;
- Adopt evidence-based guidelines for the development of dashboards, clinical reports, and patient-facing materials;
- Integrate visualisation expertise within multidisciplinary health IT and informatics teams.

The examples presented throughout this paper, particularly within the field of oncology, illustrate the transformative power of data visualisation in converting raw, heterogeneous datasets into meaningful insights that support patient-centred decisions. From visual genomic profiling for personalised therapy selection, to prognostic stratification tools like nomograms and survival curves, to interactive dashboards for longitudinal monitoring—each use case highlights the value of clear, intuitive, and functional graphics in advancing the goals of precision medicine.

In an era defined by digital transformation and data abundance, the role of visualisation is no longer ancillary. It is an essential component of clinical cognition and workflow, helping healthcare professionals to “see” what the data is telling them—and to act accordingly. Investing in well-designed visual tools is thus an investment in better, safer, and more effective care.

## Figures and Tables

**Table 1:** Advantages and limitations of pre-attentive attributes in healthcare visualisation.

Attribute	Advantages	Limitations
Colour	Draws immediate attention; easily distinguishes categories.	Can be inaccessible for colour-blind users; overuse causes confusion.
Size	Indicates importance or magnitude at a glance.	Difficult to compare precisely; may dominate space unnecessarily.
Shape	Helps differentiate types of data visually.	Too many shapes confuse interpretation.
Position	Precise comparison of values; good for alignment.	Requires careful layout to avoid clutter.
Motion	Highlights change over time or alerts.	Can be distracting; not suitable for static print media.

**Table 2:** Studies using pre-attentive strategies in clinical visualisation.

Study/Authors	Clinical Context	Pre-Attentive Strategy Used	Technical Elements	Observed Benefits
Andrade et al. [11]	Anaesthesia monitoring	Size variation in icons	Triangle icon increasing with risk	Improved event detection (14 s faster), fewer missed alarms
CBioPortal Integration Case [28,29]	Molecular Tumour Boards (MTBs)	Colour and layout for OncoPrints and mutation hotspots	Interactive heatmaps and lollipop plots	Efficient case preparation and therapy guidance
Nomogram Studies [30,31,32]	Oncology prognostic risk stratification	Position and point scales for risk prediction	Nomogram scales mapping multivariable scores to probabilities	Better individualised risk assessment and therapy planning
Seol et al. [42]	Colorectal cancer dashboard	Position, size, and icon differentiation	Multi-level dashboard with timelines and graphs	Improved workflow integration and decision accuracy
Pollack et al. [3]	Patient triage from EHR	Colour coding for severity levels	Red highlights for critical scores	Faster identification of critical cases, reduced cognitive load

## Data Availability

No new data were created or analyzed in this study.
